# Loss of function of GATA3 induces basal-like mammary tumors

**DOI:** 10.7150/thno.65796

**Published:** 2022-01-01

**Authors:** Feng Bai, Chenglong Zheng, Xiong Liu, Ho Lam Chan, Shiqin Liu, Jinshan Ma, Sijia Ren, Wei-Guo Zhu, Xin-Hai Pei

**Affiliations:** 1 Guangdong Provincial Key Laboratory of Regional Immunity and Diseases, International Cancer Center, Marshall Laboratory of Biomedical Engineering, Shenzhen University Health Science Center, Shenzhen 518060, China.; 2 Department of Pathology, Shenzhen University Health Science Center, Shenzhen 518060, China.; 3 Dewitt Daughtry Family Department of Surgery, Sylvester Comprehensive Cancer Center, University of Miami, Miami, FL 33136, USA.; 4 Xinjiang Uigur Autonomous Region People's Hospital, Xinjiang, 830001, China.; 5 Department of Biochemistry and Molecular Biology, International Cancer Center, Shenzhen University Health Science Center, Shenzhen 518060, China.; 6 Department of Anatomy and Histology, Shenzhen University Health Science Center, Shenzhen 518060, China.

**Keywords:** Gata3 loss, p18^INK4c^, mammary tumor, basal differentiation

## Abstract

**Purpose:** GATA3 is a transcription factor essential for mammary luminal epithelial cell differentiation. Expression of GATA3 is absent or significantly reduced in basal-like breast cancers. Gata3 loss-of-function impairs cell proliferation, making it difficult to investigate the role of GATA3 deficiency *in vivo*. We previously demonstrated that CDK inhibitor p18^INK4c^ (p18) is a downstream target of GATA3 and restrains mammary epithelial cell proliferation and tumorigenesis. Whether and how loss-of-function of GATA3 results in basal-like breast cancers remains elusive.

**Methods:** We generated mutant mouse strains with heterozygous germline deletion of *Gata3* in p18 deficient backgrounds and developed a Gata3 depleted mammary tumor model system to determine the role of Gata3 loss in controlling cell proliferation and aberrant differentiation in mammary tumor development and progression.

**Results:** Haploid loss of *Gata3* reduced mammary epithelial cell proliferation with induction of p18, impaired luminal differentiation, and promoted basal differentiation in mammary glands. p18 deficiency induced luminal type mammary tumors and rescued the proliferative defect caused by haploid loss of *Gata3*. Haploid loss of *Gata3* accelerated p18 deficient mammary tumor development and changed the properties of these tumors, resulting in their malignant and luminal-to-basal transformation. Expression of Gata3 negatively correlated with basal differentiation markers in MMTV-PyMT mammary tumor cells. Depletion of Gata3 in luminal tumor cells also reduced cell proliferation with induction of p18 and promoted basal differentiation. We confirmed that expression of GATA3 and basal markers are inversely correlated in human basal-like breast cancers.

**Conclusions:** This study provides the first genetic evidence demonstrating that loss-of-function of GATA3 directly induces basal-like breast cancer. Our finding suggests that basal-like breast cancer may also originate from luminal type cancer.

## Introduction

Aberrant cell differentiation has long been linked to tumorigenesis and poor differentiation, and is strongly associated with worse cancer prognosis. The molecular mechanism of how altered differentiation is linked to tumorigenesis, particularly tumor development in solid organs, is poorly understood. This study uses a well-defined *in vivo* cell differentiation system, the mammary gland, to determine how altered differentiation contributes to breast cancer.

Mammary epithelia are mainly composed of luminal and basal cells that are maintained by luminal and basal progenitors, respectively, and are believed to originate from a common mammary stem cell (MaSC) 1-5. Although little is known about the mechanisms controlling basal cell lineage differentiation, luminal cell fate determination is mainly controlled by a network of transcription factors including GATA3, ELF5, FOXA1, STAT3, and STAT5A, and deficiency or reduction of these transcription factors impairs luminal cell differentiation and mammary gland development 1, 2, 6. Clinically, breast cancer comprises three main subtypes including human epidermal growth factor receptor 2 (HER2) positive, hormone receptor [estrogen receptor (ER) and/or progesterone receptor (PGR)]-positive, and triple-negative breast cancer (TNBC) which lacks expression of ER, PGR, and HER2 7, 8. Molecularly, breast cancer is categorized into five intrinsic subtypes: basal-like (BL), HER2-enriched, luminal A, luminal B, and normal-like, each with unique biological and prognostic features 8-10. Basal-like breast cancer (BLBC) accounts for approximately 70% of TNBCs and is a leading cause of cancer deaths worldwide. The high mortality rate of BLBCs can be attributed to the aggressive, metastatic capacity of these tumors and the limited number of effective therapeutic options 7, 8. BLBCs are heterogeneous and contain several distinct cell types including cells that express luminal biomarkers 11-13. It was recently suggested that BRCA1 deficient BLBCs may originate from luminal progenitors 14-17. Yet, two elusive questions remain: how BLBCs develop and whether loss-of-function of transcription factors essential for luminal cell differentiation contributes to basal differentiation during mammary tumorigenesis and progression.

GATA3 has dual roles in both normal and tumor development. It plays a critical role in the development of the nervous system, mammary gland, parathyroid glands, kidney, inner ear, skin, and lymphoid cell lineage 18-23. Germline mutations of *GATA3* in humans are associated with the congenital hypoparathyroidism-deafness-renal disease (HDR) syndrome 24, 25. Somatic mutations of *GATA3* have been detected in ~15% of breast cancers and is one of the top three genes mutated in >10% of all breast cancers. Interestingly, most breast cancers with *GATA3* mutation are luminal type cancers that retain GATA3 expression 8, 26, and high GATA3 expression predicts better survival 1, 27. However, *GATA3* is often silenced by DNA methylation 28, 29 and its expression is lost or significantly reduced in BLBCs 8, 27, 30, 31. It has not been determined if loss-of-function of GATA3 induces BLBCs.

Gata3 is required for mammary luminal epithelial differentiation and mammary gland development 19, 20. Germline or epithelium-specific deletion of *Gata3* in mice causes early lethality or severe growth defects 18-20, 32, making it difficult to study its loss-of-function in mammary tumorigenesis, also suggesting that overcoming growth defects is a necessary step for the development of tumors initiated by GATA3 reduction or loss. Overexpression of GATA3 suppresses epithelial-mesenchymal transition (EMT) in cancer cell lines 33, 34 and loss of Gata3 in transgenic mice stimulates mammary luminal tumor progression 35, 36. However, due to the inability to tolerate Gata3 loss in differentiated luminal tumors in transgenic mice^35^, it remains elusive whether and how Gata3 loss-of-function alters the fate of luminal cells and induces aberrant differentiation, stimulating mammary tumor progression.

Not until recently has the function of GATA3 in regulating cell proliferation been reported. Loss of Gata3 results in impaired cell cycle entry and proliferation of hematopoietic stem cells (HSC) 37, though the discrepant finding that deletion of *Gata3* enhances self-renewal of HSCs without affecting the cell cycle has also been observed 38. We, and others, demonstrated that loss of Gata3 impairs T cell proliferation 39-41 and reduces the proliferation of mammary luminal epithelial cells 27 and T cells 40, 41 with induction of cell cycle inhibitor, *p18^Ink4c^* (*p18*). p18 is a member of the INK4 family that inhibits CDK4 and CDK6, whose activation by mitogen-induced D-type cyclins lead to phosphorylation and functional inactivation of RB, p107, and p130. Loss-of-function of the INK4-cyclin D/CDK4/6-RB pathway is a common event in variety of cancers including breast cancer 42 and *p18* expression is significantly lower in human luminal breast cancers 8, consistent with our finding that loss of p18 in mice induces luminal mammary tumors 27. Importantly, depletion of both Rb and p107 in mice also results in luminal type tumors 43. These observations suggest a role of the p18-Rb pathway in controlling luminal tumorigenesis.

Prompted by the finding that p18 is a downstream target of GATA3 and restrains mammary epithelial cell (MEC) proliferation and tumorigenesis 27, we hypothesize that p18 deficiency may rescue GATA3 deficiency impaired MEC proliferation, allowing us to determine the role of Gata3 loss in controlling cell fate during mammary tumorigenesis. In the present study, we generated mutant mouse strains with heterozygous germline deletion of *Gata3* in p18 deficient backgrounds and developed a Gata3 depleted mouse mammary tumor model system to determine the function and mechanism of Gata3 loss in controlling cell proliferation and aberrant differentiation in mammary tumor development and progression.

## Methods

### Mice, histopathology, and immunohistochemistry

The generation of *p18^-/-^, p18^+/-^, Gata3^+/-^, p18^+/-^;Gata3^+/-^, and p18^-/-^;Gata3^+/-^* mice was previously described 17, 27, 41, 44. NOD-Prkdcem26Cd52Il2rgem26Cd22/Nju (NCG) and FVB/NJGpt-Tg(MMTV-PyMT)/Gpt were purchased from GemPharmatech (Nanjing, China). The Institutional Animal Care and Use Committee at the University of Miami and Shenzhen University approved all animal procedures. Histopathology and immunohistochemistry (IHC) were performed as previously described 17, 27, 44. The primary antibodies used were: E-cadherin (E-Cad) (BD Biosciences), Ck5 (Covance), Ck8 (American Research Products), Ck14 (Thermal Scientific), eGFP (GeneTex), GATA3 (Santa Cruz), SMA (Cell Signaling), and Ki67 (Abcam). Immunocomplexes were detected using the Vectastain ABC alkaline phosphatase kit according to the manufacturer's instructions (Vector Laboratories), or using FITC- or rhodamine-conjugated secondary antibodies (Jackson Immunoresearch). IHC results were quantified using H-score as previously described 45, 46.

### Normal mammary and tumor cell preparation, FACS analysis, colony-formation assay, cell culture, and knockdown of GATA3

Tumor-free mammary glands were isolated from mice at the indicated ages and genotypes, the tissue was processed, mammary cell suspensions were prepared, and FACS analysis and colony-formation assays were performed as previously described 3, 5, 27. Mammary tumors were dissected from female mice and tumor cell suspensions were prepared as previously described 17, 27, 44. Primary mammary tumor cells isolated from female MMTV-PYMT mice were cultured in either MEC medium [10% FBS (Gibco), 10 ng/ml EGF, 1 μg/ml hydrocortisone] or MM+ medium [2% FCS (Gibco), 1% BSA]. T47D cells were cultured per ATCC recommendations. For knockdown of Gata3 in MMTV-PYMT tumor cells, cells were infected with psi-LVRU6GP-control, psi-LVRU6GP-Gata3-a, or psi-LVRU6GP-Gata3-c (GeneCopoeia, Guangzhou, China), then selected with puromycin. eGFP positive cells were the cells successfully infected with psi-LVRU6GP virus. For knockdown of GATA3 in human tumor cells, cells were infected with pGIPZ-empty, pGIPZ-shGATA3-E9, and pGIPZ-shGATA3-A12 as previously described 44.

### Transplantation model of mammary tumors

For the transplantation of primary MMTV-PyMT mammary tumor cells,1 x 10^6^ cells infected with psi-LVRU6GP-control or psi-LVRU6GP-Gata3-c and selected with puromycin were inoculated into the left and right inguinal mammary fat pads (MFPs) of 6-week-old female NCG mice, respectively. Eight weeks after transplantation, animals were euthanized and mammary tumors were dissected for histopathological, immunohistochemical, and biochemical analyses. For the transplantation of primary *p18^mt^ (p18^+/-^ and p18^-/-^ )* and* p18^mt^;Gata3^+/-^* (*p18^+/-^;Gata3^+/-^* and *p18^-/-^;Gata3^+/-^* ) mammary tumor cells, cells were inoculated into the left and right inguinal MFPs of female NCG mice, respectively, along with subcutaneous implantation of estrogen pellets. Eight weeks after transplantation, animals were euthanized and tumors were analyzed by histopathology and immunohistochemistry.

### Western blot and qRT-PCR

Tissue and cell lysates were prepared as previously reported 44. Primary antibodies used were as follows: HSP90, GAPDH (Ambion), E-Cad (Cell Signaling), GATA3 (HG3-31, Santa Cruz). For qRT-PCR, total RNA was extracted using the RNeasy kit (Qiagen) according to the manufacturer's protocol and cDNA was generated using the Omniscript RT Kit (Qiagen). qRT-PCR was performed as reported 44. Primers used are listed in Table S1.

### Meta-analysis of gene expression data sets

The correlation of GATA3 mRNA and protein expression with molecular subtypes or major subtypes was analyzed in TCGA or in Clinical Proteomic Tumor Analysis Consortium (CPTAC) breast cancer samples 47, 48. The human breast cancer gene expression miner v4.6 dataset with 11,359 samples (http://bcgenex.ico.unicancer.fr/BC-GEM/GEM-requete.php) 48 was analyzed for correlation of expression of *GATA3* with genes associated with basal differentiation.

### Statistical analysis

The survival rate was calculated by the Kaplan-Meier method. Mantel-Cox log-rank tests were applied to compare the survival difference and obtained p values. All data are presented as the mean ±SD for at least three repeated individual experiments for each group. Quantitative results were analyzed by two-tailed Student's t-test. P < 0.05 was considered statistically significant.

## Results

### Deficiency of Gata3 reduces MEC proliferation with induction of p18

**Figure 1:**
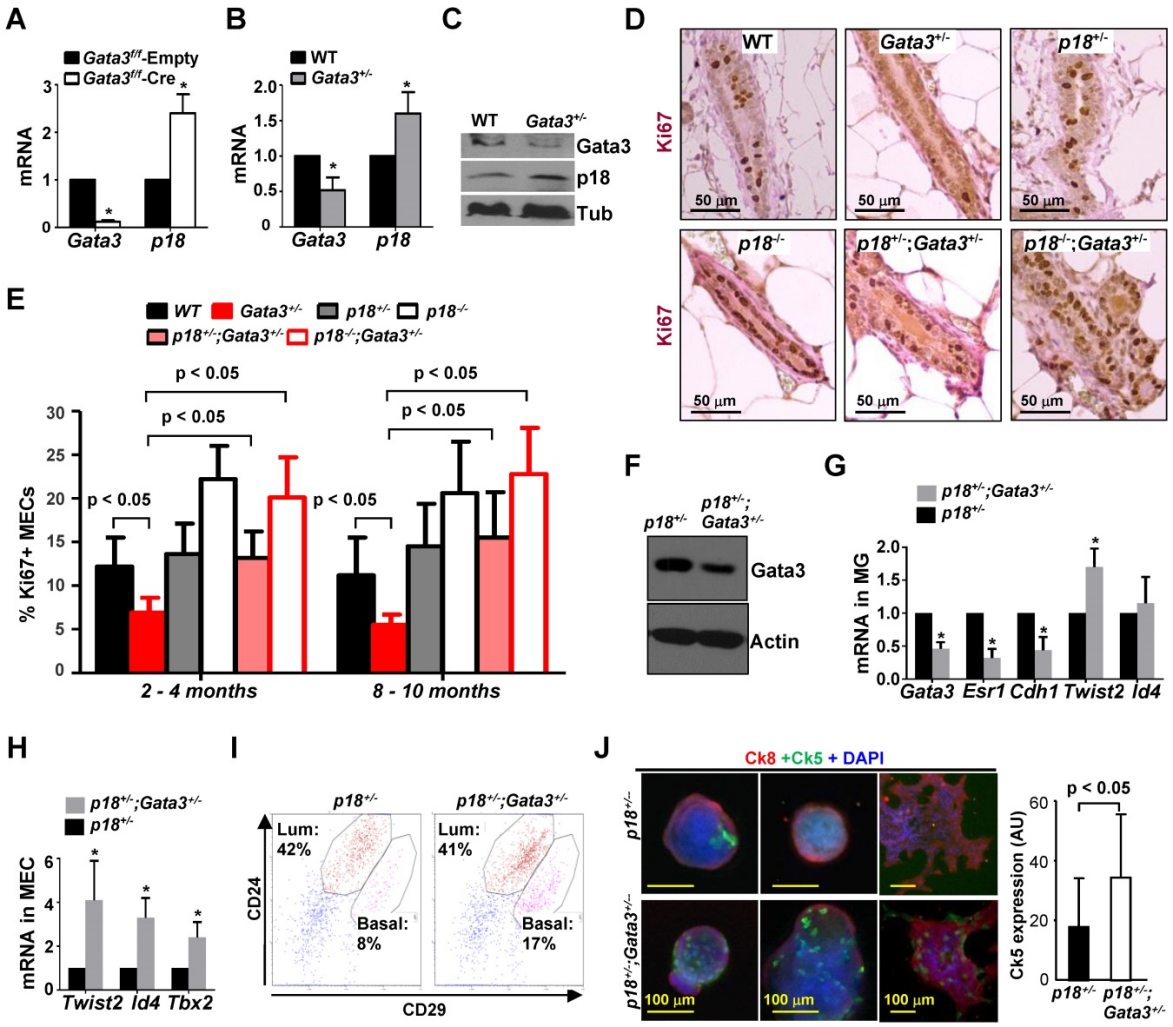
** Gata3 deficiency promotes basal differentiation and reduces proliferation in MECs, and p18 deficiency rescues proliferative defects caused by *Gata3* heterozygosity.** (A) MECs isolated from 3-month-old female *Gata3*^f/f^ mice were infected with pMX-Cre (Cre) and pMX-Empty (Empty) then selected with puromycin for 3 days. mRNA was extracted and analyzed. (B, C) RNA and protein lysates extracted from WT and *Gata3*^+/-^ MECs were analyzed by qRT-PCR (B) and western blot (C). Data in (A) and (B) represent the mean ± SD from triplicates of two independent primary cell lines of each genotype. The asterisk (*) denotes a statistical significance between WT and *Gata3*^+/-^ or Cre and Empty samples as determined by T-test. (D) Representative mammary tissues from 8-10-month-old mice were analyzed by immunohistochemistry with Ki67. (E) The percentages of Ki67-positive cells were calculated from cells situated in clear duct/gland structures from 2-4 month and 8-10-month-old mice, respectively. Results represent the mean ± SD of three animals per group. (F-H) Tumor-free mammary glands (MG, F, G) and mammary epithelial cells (MEC, H) from 2-4-month-old mice were analyzed by western blot (F) and qRT-PCR (G, H). Data are expressed as the mean ± SD from triplicates of each of three separate mice (G) or of four different lines of MECs (H). The asterisk (*) denotes a statistical significance from *p18*^+/-^;*Gata3*^+/-^ and *p18*^+/-^ samples determined by T-test. (I) Representative mammary cells from 2-4-month-old mice were analyzed by flow cytometry. (J) Freshly isolated mammary cells from 2-4-month-old mice were cultured in Matrigel-coated 24 well plates. Nine days after culture, the colonies were immunostained with Ck8 and Ck5 (left panel). Ck5 expression in colonies was quantified by ImageJ software (right panel). The assay was performed in triplicate for each animal. The bar graphs represent the mean ± SD of two animals per group.

We previously demonstrated that deletion of *Gata3* in mouse mammary gland *in vivo* promotes the expression of p18 27. To confirm the role of Gata3 in regulating p18 in MECs, we infected *Gata3^f^*^/f^ MECs with pMX-Cre and found that deletion of *Gata3*, as expected, drastically enhanced p18 expression (Figure 1A). We previously detected expression of Gata3 in both luminal and MaSC-enriched basal epithelial cells, though GATA3 levels in the former is more abundant than in the latter. However, Gata3 is hardly detectable in stromal cells 27, suggesting it may function in both luminal and MaSC-enriched basal cells. To determine the effect of Gata3 deficiency in regulating all mammary cell lineages in an unbiased manner, we generated heterozygous germline *Gata3*^+/-^ mutant mice by crossing *Gata3*^f/+^ mice with BALB/c-CMV-cre mice, a germline “Cre-deleter” strain, as we previously described 41. We confirmed reduced Gata3 mRNA and protein levels in *Gata3*^+/-^ mammary glands as well as in thymocytes and splenocytes 41. We detected increased p18 expression in *Gata3*^+/-^ mammary glands when compared with WT counterparts (Figure 1B, C, Figure S1A). MEC proliferation, as evidenced by Ki67 staining, in *Gata3*^+/-^ mice relative to WT animals was significantly reduced (6.9% ± 1.7% vs. 12.2% ± 3.3%, p < 0.05 at 2-4 months of age, 5.5% ± 1.2% vs. 11.2% ± 4.3%, p < 0.05 at 8-10 months of age; Figure 1D, E, and Figure S1B). Consistent with our previous finding derived from *Gata3*^f/f^;WAP-cre mice 27, these results indicate that haploid loss of *Gata3* caused by heterozygous germline deletion of *Gata3* also reduces MEC proliferation that is associated with induction of p18, further confirming that p18 is a downstream target of Gata3 in restraining MEC proliferation.

### p18 deficiency rescues proliferative defects of Gata3 deficient MECs and haploid loss of *Gata3* promotes basal differentiation

Identification of p18 as a downstream target of Gata3 in restraining mammary epithelial cell proliferation prompted us to hypothesize that p18 deficiency may rescue mammary growth defects caused by *Gata3* deletion, allowing us to investigate the role of Gata3 loss-of-function in controlling cell fate during mammary tumor development and progression. We crossed *p18*^-/-^ mice with *Gata3* mutants and generated *p18^-/-^;Gata3^+/-^* and *p18^+/-^;Gata3^+/-^* mice in Balb/c-B6 mixed background. We found that Ki67 positive MECs from *p18^-/-^;Gata3^+/-^* mice were comparable with those from *p18^-/-^* mice (20.1% ± 4.6% vs. 22.2% ± 3.8% at 2-4 months, 22.8% ± 5.3% vs. 20.6% ± 5.9% at 8-10 months)*,* and* p18^+/-^;Gata3^+/-^* comparable with *p18^+/-^* (13.2% ± 3.0% vs. 13.6% ± 3.5% at 2-4 months, 15.5% ± 5.2% vs. 14.5% ± 4.9% at 8-10 months). However, the number of Ki67 positive MECs in both *p18^-/-^;Gata3^+/-^* and *p18^+/-^;Gata3^+/-^* mice were significantly more than in *Gata3^+/-^* animals (20.1% ± 4.6% in *p18^-/-^;Gata3^+/-^* and 13.2% ± 3.0% in *p18^+/-^;Gata3^+/-^* vs. 6.9% ± 1.7% in *Gata3^+/-^* at 2-4 months, 22.8% ± 5.3% in *p18^-/-^;Gata3^+/-^* and 15.5% ± 5.2% in *p18^+/-^;Gata3^+/-^* vs. 5.5% ± 1.2% in *Gata3^+/-^* at 8-10 months. (Figure 1D, E, and Figure S1A). These results suggest that haploid or complete loss of *p18* rescues the proliferative defect induced by haploid loss of *Gata3* in MECs, and p18 deficiency is required for Gata3 deficient MEC proliferation. We then confirmed decreased Gata3 expression in *p18^+/-^;Gata3^+/-^* mammary tissues relative to *p18^+/-^* tissues, and determined the differentiation changes in Gata3 deficient MECs (Fig. 1F, G). We found that the expression of *Esr1* (encoding ERα) and *Cdh1* - genes associated with luminal differentiation - in* Gata3^+/-^* and *p18^+/-^;Gata3^+/-^* mammary glands was reduced relative to WT and *p18^+/-^* glands (Fig. 1G and data not shown), confirming that Gata3 deficiency impairs luminal differentiation 19, 20. Notably, the expression of *Twist2*, *Id4* and *Tbx2* - transcription factors associated with basal differentiation 44, 49, 50 - was enhanced in* Gata3^+/-^* and *p18^+/-^;Gata3^+/-^* mammary glands and MECs relative to WT and *p18^+/-^* counterparts (Fig. 1G, H, and data not shown). We did not detect significant changes in luminal progenitor (LP) -enriched luminal populations in *p18^+/-^;Gata3^+/-^* MECs by FACS analysis (CD24^+^CD29^low^, Fig. 1I) compared to *p18^+/-^* MECs, suggesting that haploid loss of *Gata3* is insufficient to impact this population. However, MaSC-enriched basal populations in *p18^+/-^;Gata3^+/-^* MECs were clearly increased (CD24^+^CD29^high^, Fig. 1I) relative to *p18^+/-^* MECs. We performed colony formation assays and found that *p18^+/-^;Gata3^+/-^* MECs produced significantly more basal colonies than* p18^+/-^* (Fig. 1J, Figure S1C), further supporting a function of Gata3 in suppressing basal colony formation. In sum, these results indicate that haploid loss of *Gata3* promotes basal differentiation in MECs.

### Haploid loss of *Gata3* in p18 deficient mice convert luminal type tumors into basal-like tumors

**Figure 2:**
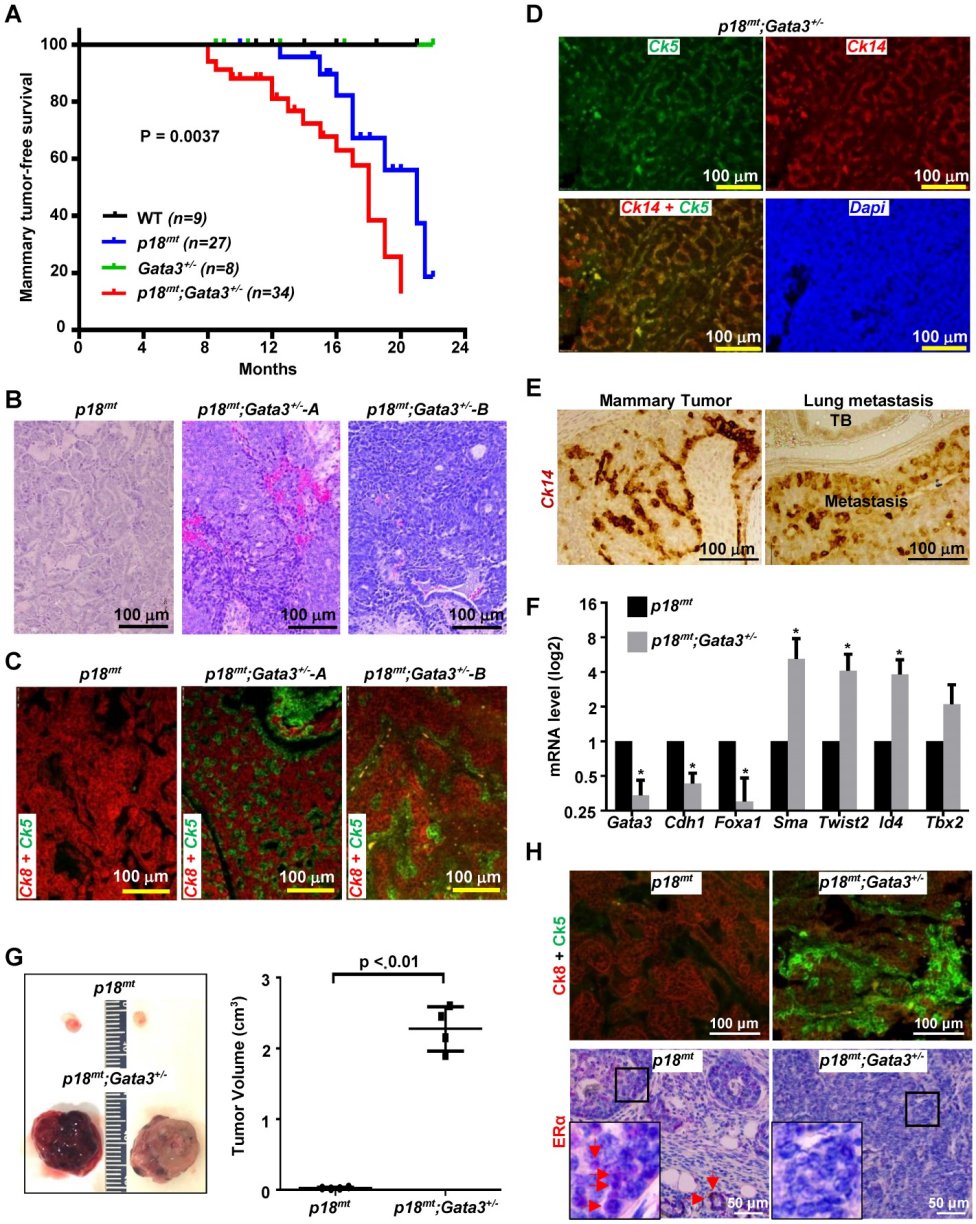
***Gata3* heterozygosity in p18 deficient mice induces basal-like mammary tumors.** (A) Mammary tumor-free survival of mice in Balb/c-B6 mixed background. Mammary tumor-free median survival was 18 months in *p18^mt^;Gata3^+/-^* mice and 21 months in *p18^mt^* mice. The *p18^mt^* mouse group includes eight *p18^+/-^* and nineteen *p18^-/-^* mice, and the *p18^mt^;Gata3^+/-^* mouse group includes ten *p18*^+/-^;*Gata3*^+/-^ and twenty-four *p18*^-/-^;*Gata3*^+/-^ mice. (B-D) Representative H&E (B), and IF staining (C, D) of primary mammary tumors developed in mice with the indicated genotypes. (E) A representative *p18^mt^;Gata3^+/-^* spontaneous mammary tumor and lung metastatic lesion were analyzed by IHC with an antibody against Ck14. TB, Terminal Bronchiole. (F) RNA extracted from representative mammary tumors of the indicated genotype were analyzed. Results represent the mean ± SD of three tumors from individual animal per group. The asterisk (*) denotes a statistical significance from *p18^mt^;Gata3^+/-^* and *p18^mt^* samples determined by T-test. (G) Primary *p18^mt^;Gata3^+/-^* (5 x 10^5^) and *p18^mt^* (5 x 10^6^) mammary tumor cells were transplanted into the left and right inguinal MFPs of female NCG mice, respectively. Gross appearance and volume of mammary tumors regenerated in 8 weeks were analyzed. Data are represented as mean ± SD for tumors in each group (n = 4). (H) Mammary tumors formed by transplantation of *p18^mt^;Gata3^+/-^* and *p18^mt^* tumor cells in (G) were immunostained with antibodies against Ck5, Ck8, and ERα. The boxed areas were enlarged in the insets. Representative ERα-positive tumor cells and luminal epithelial cells in tumor-free glands are indicated by red arrows. Note that ERα-positive cells were mainly detected in *p18^mt^* tumors, but barely observed in *p18^mt^;Gata3^+/-^* tumors whereas Ck5-positive cells were mainly detected in* p18^mt^;Gata3^+/-^* tumors and hardly found in *p18^mt^* tumors.

We followed tumor development in WT and mutant mice with Balb/c-B6 mixed background. Because both *p18*^+/-^ and *p18*^-/-^ mice spontaneously develop luminal type mammary tumors 17, 27, and either haploid or complete loss of *p18* rescues the proliferative defect of *Gata3*^+/-^ MECs, we combined *p18*^-/-^ and *p18*^+/-^ mice as the *p18*^mt^ group, and *p18*^-/-^;*Gata3*^+/-^ and *p18*^+/-^;*Gata3*^+/-^ mice as the *p18*^mt^;*Gata3*^+/-^ group. Starting from as early as 8 months, *p18*^mt^*;Gata3*^+/-^ mice (n *=* 34) developed mammary tumors whereas *p18^mt^* mice (n = 27) developed mammary tumors after 12.5 months. However, no WT (n = 9) nor *Gata3*^+/-^ (n = 8) mice did within the same time period. The mammary tumor-free survival was reduced from a mean age of 21 months in *p18*^mt^ mice to 18 months in *p18^mt^;Gata3^+/-^* mice (Figure 2A). These results demonstrate that overcoming growth defects, e.g., deficiency of p18, is required for Gata3 deficient MEC transformation and tumor development and that haploid loss of *Gata3* in p18 deficient mice accelerates mammary tumorigenesis.

Further characterization of mammary tumors revealed that 75% (n = 8) of *p18*^mt^ mammary tumors were, as we previously reported 27, well-differentiated, ER and Ck8 positive, luminal type tumors (Table 1, Figure 2B-E). On the other hand, 82% (n = 17) of *p18^mt^;Gata3^+/-^* mammary tumors were highly heterogeneous, poorly-differentiated, ER negative, Ck5, Ck14 or SMA positive basal-like tumors (Table 1, Figure 2B-E). Relative to *p18^mt^* mammary tumors, *p18^mt^;Gata3^+/-^* tumors exhibited reduced expression of genes associated with luminal differentiation and enhanced expression of genes associated with basal differentiation (Figure 2F). Importantly, 29% (5 of 17) of *p18*^mt^;*Gata3*^+/-^ and none (0 of 8) of *p18*^mt^ mammary tumors metastasized to the lung, and all lung metastases were positive for mammary basal marker, Ck14 (Fig. 2E). These data indicate that haploid loss of *Gata3* by heterozygous germline deletion of *Gata3*, although insufficient to induce mammary tumors alone, significantly changes the properties of mammary tumors induced by *p18* deficiency, resulting in their malignant and luminal-to-basal-like transformation.

We transplanted primary tumor cells into MFPs of mice and found that all mice that received 5 x 10^6^ *p18^mt^* tumor cell transplants produced small tumors (26 ± 11 mm^3^ in size) in 8 weeks. Regenerated* p18^mt^* mammary tumors, like primary *p18^mt^* tumors, were well differentiated, positive for Ck8 and ERα, and negative or nearly undetectable for Ck5 and Ck14. In the same time period, all mice that received 5 x 10^5^ *p18^mt^;Gata3^+/-^* tumor cell transplants, 1/10 the number of cells in comparison with* p18^mt^* transplants, developed large mammary tumors (2,275 ± 312 mm^3^). Pathological and IHC analysis revealed that, like primary *p18^mt^;Gata3^+/-^* mammary tumors, regenerated *p18^mt^;Gata3^+/-^* mammary tumors were poorly differentiated, positive for Ck5 and Ck14, and negative for ERα. (Figure 2G, H, and data not shown). These results confirm that in mammary tumor cells, *Gata3* deficiency not only enhances the potential for tumor initiation but also promotes luminal-to-basal differentiation.

### Expression of Gata3 negatively correlates with basal differentiation markers in mammary tumor cells

**Figure 3:**
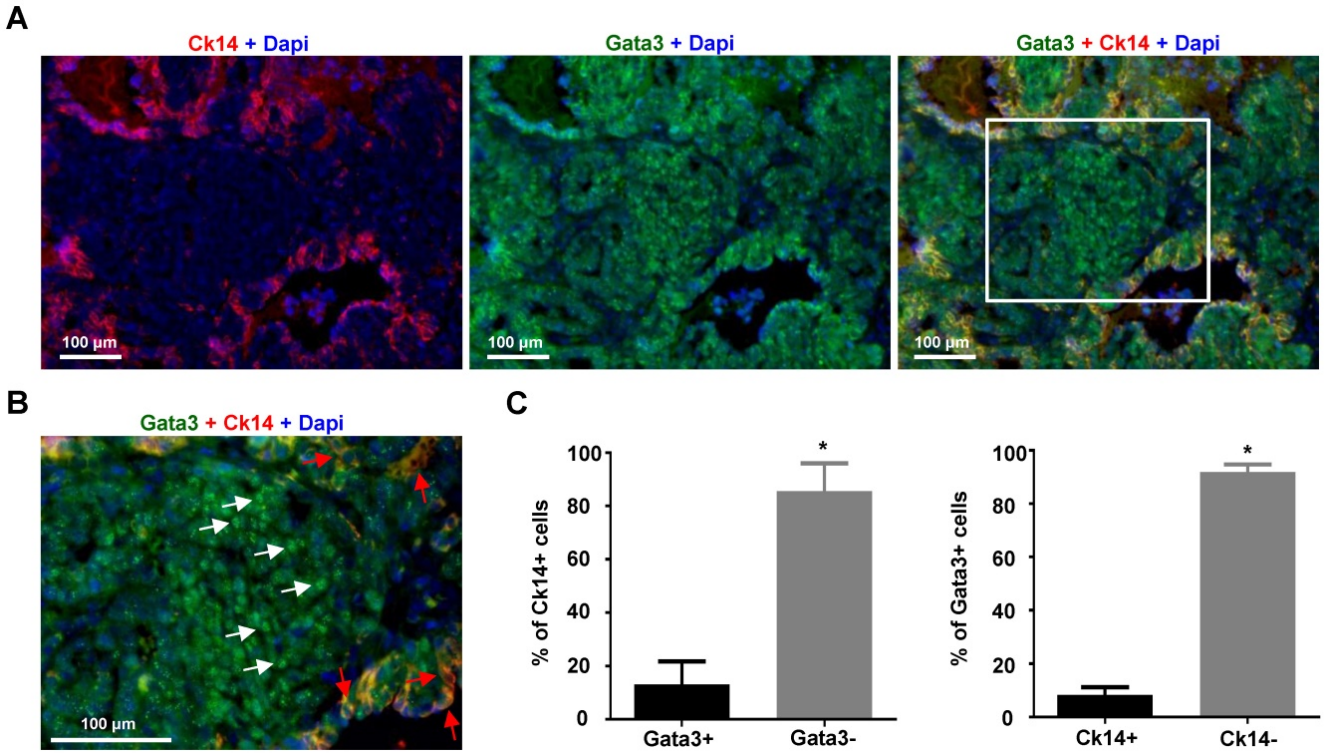
** The expression of Gata3 is negatively correlated with Ck14 in MMTV-PyMT mammary tumor cells.** (A) Representative immunofluorescent staining of primary MMTV-PyMT mammary tumors with antibodies against Gata3 and Ck14. (B) Enlarged view of the boxed area in (A). Gata3 (white arrows) and Ck14 (red arrows) positive cells are indicated. (C) Quantification of Gata3 and Ck14 positive cells. The percentages of Ck14+ or Ck14- and Gata3+ or Gata3- cells were calculated from Gata3+Dapi+ and Ck14+Dapi+ cells, respectively. The results represent the mean ± SD of three individual tumors. At least 500 Ck14+ and Gata3+ cells were counted for each tumor. The asterisk (*) denotes a statistical significance from two group samples determined by T-test.

To determine whether loss of expression of *GATA3* is associated with basal differentiation in established mammary tumors, we took advantage of MMTV-PyMT mice which develop spontaneous mammary tumors that have been well characterized as luminal B type tumors with a small number of Ck14 positive basal cells 35, 49, 51, 52. We carefully analyzed MMTV-PyMT mammary tumors which developed in 8-14 weeks. We found that ~86% of Ck14 positive cells did not express nuclear GATA3 and only ~13% of Ck14 positive cells co-expressed Gata3 (Figure 3A-C). Consistently, ~92% of Gata3 positive cells were negative for Ck14 and ~8% Gata3 positive cells were Ck14 positive. Importantly, in nearly all Ck14 and Gata3 double positive cells, Gata3 lost its nuclear localization, indicative of loss of Gata3 transactivation activity (Figure 3B, C). These results suggest that expression of Gata3 is negatively correlated with the basal marker CK14 in mammary tumor cells.

### Generation of a Gata3 positive luminal type tumor model system

Due to slow proliferation *in vitro*, terminal differentiation status in nature (see below), as well as long latency for tumor initiation and exogenous estrogen dependent tumor growth *in vivo*, widely used human luminal type breast cancer cell lines such as MCF7 and T47D are not appropriate for investigating luminal tumor cell reprogramming in tumorigenesis and progression. To build a murine model system for investigating the role of Gata3 in controlling basal differentiation in luminal tumor cells, we screened 19 spontaneous mammary tumors developed in PyMT mice by western blot and IHC. We noticed that the tumors expressed distinct levels of Gata3 (Figure S2A). We chose 10 individual tumors with various levels of Gata3 and cultured them for further characterization. We found that after two weeks in culture, more than 90% of cells derived from tumors with high levels of Gata3 (e.g., B tumor cell line) belonged to a CD24^+^CD29^low^ population in two different culture media whereas cells derived from tumors with low levels of Gata3 (e.g., C tumor cell line) contained a CD24^-^CD29^low^ population in FBS high medium (MEC medium) or a mixture of CD24^-^CD29^low^ and CD24^+^CD29^low^ populations in FBS low medium (MM+ medium). After 6 weeks of culture in either FBS high or low medium, more than 90% of cells with high levels of GATA3 retained their CD24^+^CD29^low^ feature, however, nearly all cells with low levels of Gata3 were CD24^-^CD29^low^ (Figure S2B). These results suggest that in our cell culture system, primary tumor cells with high levels of Gata3 maintained their CD24^+^CD29^low^ feature which is, as previously reported 3, 27, 53, a characteristic of luminal tumor cells by FACS analysis. Importantly, this CD24^+^CD29^low^ feature is a typical characteristic of primary and regenerated MMTV-PyMT mammary tumor cells 35. We confirmed that after long term culture (at least 6 months), tumor cells derived from cells with high levels of Gata3 maintained their expression of Gata3 and E-Cad (encoded by *Cdh1*) (Figure S2C, and data not shown). We then transplanted these tumor cells (e.g., B tumor cell line) into MFPs of NCG mice. We confirmed that the newly regenerated tumors expressed comparable levels of Gata3 and E-Cad as the primary mammary tumor and that individual regenerated tumors also expressed comparable levels of Gata3 and E-Cad, indicating that the system is stable in maintaining Gata3 expression in cells and regenerated tumors (Figure S2D, E). In sum, we successfully developed a murine luminal type mammary tumor system in which mammary tumor cells express high levels of Gata3 and are capable of generating Gata3 positive luminal tumors once transplanted into the MFPs of mice.

### Depletion of Gata3 in luminal tumor cells promotes basal-like differentiation, induces p18, and reduces cell proliferation

**Figure 4:**
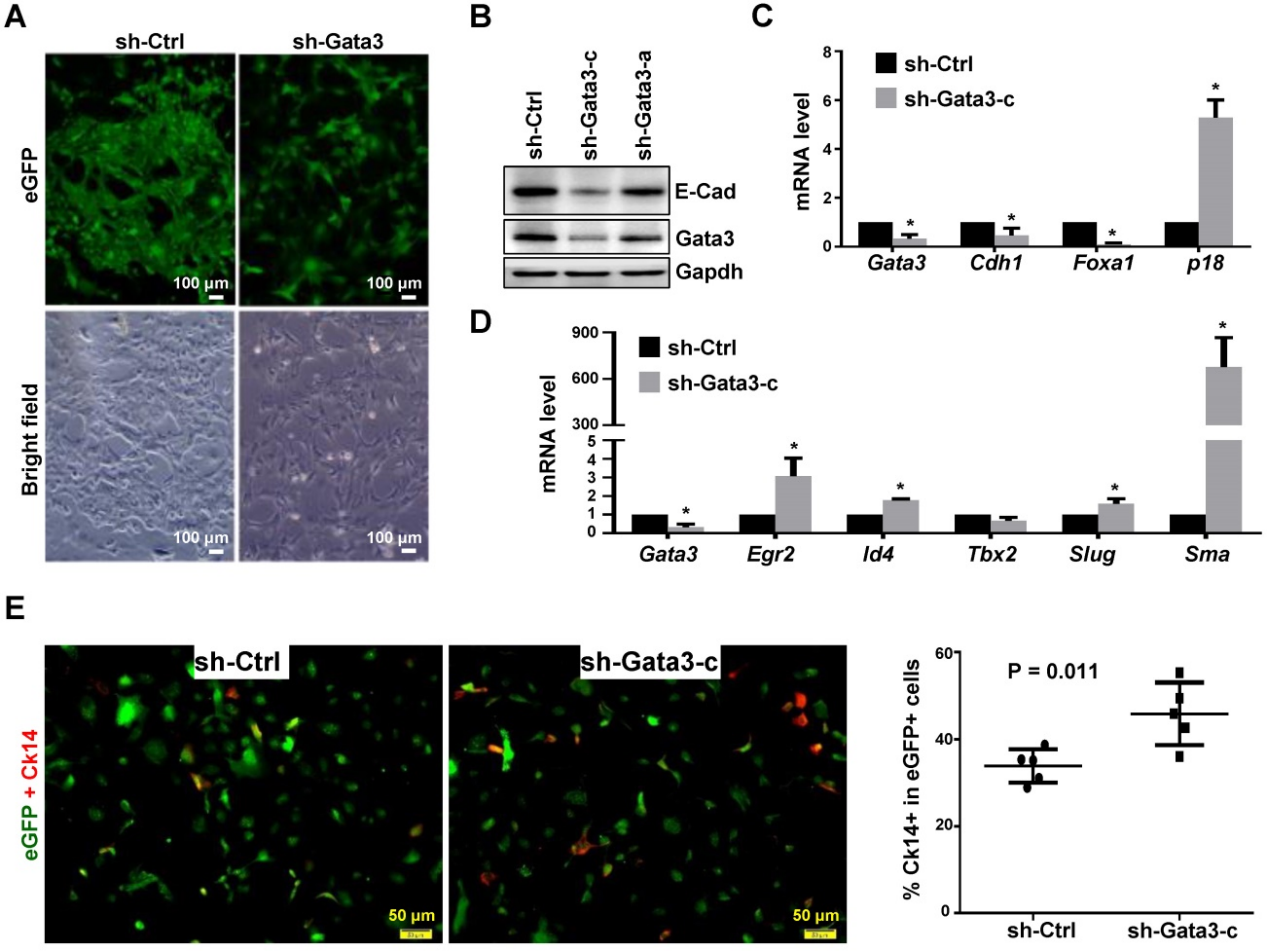
**Depletion of Gata3 in luminal tumor cells promotes basal-like differentiation *in vitro*.** (A, B) Luminal mammary tumor cells from MMTV-PyMT mice were infected with psi-LVRU6GP-control (sh-Ctrl) or psi-LVRU6GP-Gata3 targeting different sequences of mouse *Gata3* (sh-Gata3-a and sh-Gata3-c), selected with puromycin, and analyzed by phase-contrast and fluorescence microscope (A) or western blot (B). Note that sh-Ctrl cells exhibited cobblestone morphology and close-contact with neighboring cells at cell junctions whereas sh-Gata3 cells displayed an elongated and spiky appearance and were isolated. (C, D) MMTV-PyMT luminal tumor cells infected with sh-Ctrl and sh-Gata3-c were analyzed by qRT-PCR. Data represent the mean ± SD from triplicates of primary tumor cells of each group. The asterisk (*) denotes a statistical significance from sh-Ctrl and sh-Gata3-c samples determined by student's t-test. (E) Representative immunofluorescent staining analysis of the MMTV-PyMT luminal tumor cells infected with sh-Ctrl and sh-Gata3-c. Cells were immunostained with antibodies against eGFP (green) and Ck14 (red). Percentage of Ck14 positive cells in the eGFP positive cell population was calculated. Data represent the mean ± SD from more than 500 eGFP positive cells in five randomly selected fields for each group of the two independent experiments.

To determine whether and how depletion of GATA3 in established mammary tumor cells promotes basal differentiation and impacts tumor progression, we took advantage of the newly established Gata3 positive murine luminal type tumor model system. We knocked down Gata3 in Gata3 positive luminal tumor cells and noticed that Gata3 knockdown (KD) cells displayed an elongated and spiky appearance with isolated and spreading features, while control cells exhibited a typical cobblestone morphology and maintained close contact with neighboring cells (Figure 4A, B). KD of Gata3 induced the expression of p18 and reduced cell proliferation (Figure 4C, and data not shown), which is consistent with our previous finding that p18 is a downstream target of GATA3 constraining luminal cell proliferation. In addition to the reduced expression of luminal differentiation markers such as *E-cad* and *Foxa1*, depletion of Gata3 significantly enhanced the expression of basal differentiation markers and transcription factors, such as *Sma*, *Egr2*, *Slug*, and *Id4* (Figure 4C, D). We determined basal-like differentiation by Ck14 staining and found that the percentage of Ck14 positive cells in the Gata3-depleted group was significantly higher than in the control group (Figure 4E, Figure S3). We knocked down GATA3 in the human luminal breast cancer cell line, T47D, and again found that the expression of genes associated with luminal differentiation, such as *CDH1* and *ESR1*, was significantly downregulated. However, the expression of genes associated with basal differentiation such as *TWIST2*, *EGR2*, and *SMA*, was not drastically upregulated (Figure S4). The reason the expression of basal genes in T47D cells was not clearly enhanced is likely because T47D cells are in terminal differentiation status after long-term and multi-passage culture. It was indeed demonstrated that T47D cells are terminally differentiated and resistant to further differentiation 54. Together, these results indicate that depletion of Gata3 in luminal tumor cells reduces luminal differentiation but stimulates basal-like differentiation *in vitro*.

**Figure 5:**
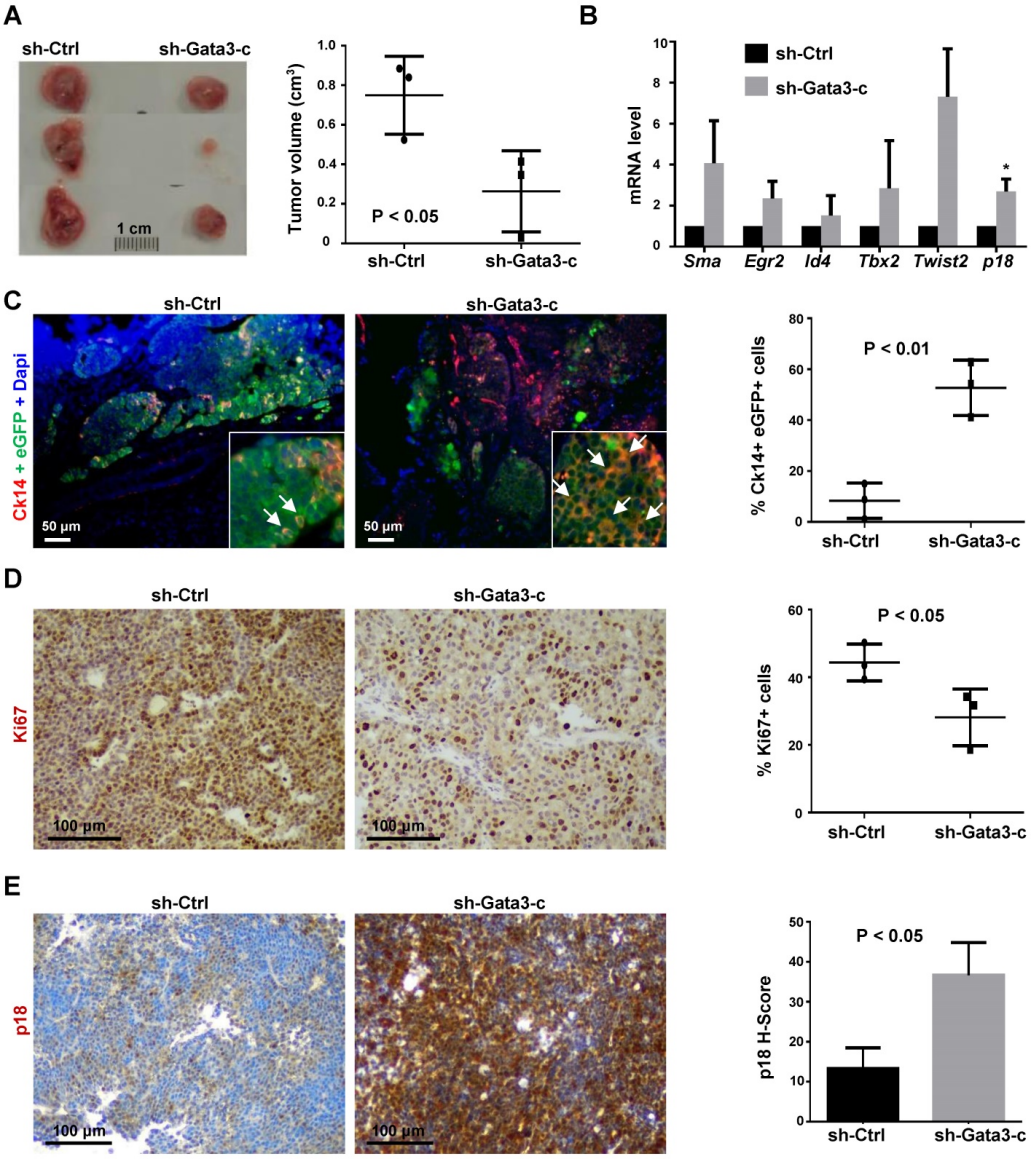
** Depletion of Gata3 converts luminal type mammary tumors into basal-like tumors.** (A) MMTV-PyMT luminal tumor cells infected with sh-Ctrl and sh-Gata3-c were transplanted into the mammary fat pads (MFPs) of female NCG mice. Gross appearance of tumors formed 8 weeks after transplantation is shown (left panel) and tumor volumes are plotted (right panel). Data represent the average tumor volumes ±SD of three tumors from individual animals per group. (B) mRNA levels of the indicated genes in regenerated tumor tissues were analyzed by qRT-PCR. Results represent the mean ± SD of three tumors from individual animals per group. The asterisk (*) denotes a statistical significance from sh-Ctrl and sh-Gata3-c samples determined by student's t-test. (C, D, E) Mammary tumors formed by transplantation of MMTV-PyMT luminal tumor cells stably expressing sh-Ctrl or sh-Gata3-c were immunostained with antibodies against eGFP and Ck14 (C), Ki67 (D), or p18 (E). The percentages of Ck14-positive cells were calculated from eGFP-positive cells, and the results represent the mean ± SD of two individual tumors per group. Ck14 and eGFP double positive cells are indicated by white arrows (C). The percentages of Ki67-positive cells were quantitated in five randomly selected fields in sections, and the results represent the mean ± SD of three animals per group (D). The H-scores for p18 were calculated. The results represent the mean ± SD of three individual tumors per group (E).

We then transplanted MMTV-PyMT luminal tumor cells into MFPs of NCG mice and unexpectedly found that *Gata3* depleted cells resulted in a significantly smaller tumor than control cells (Figure 5A). Consistent with the data derived from *in vitro* analyses, tumors generated from Gata3 depleted cells also expressed higher levels of *p18* mRNA and genes associated with basal differentiation (Figure 5B). The percentage of Ck14 positive cells in Gata3 depleted tumor cells was significantly higher than in control tumor cells (Figure 5C, Figure S5), confirming that depletion of Gata3 promotes basal-like differentiation in luminal tumor cells *in vivo*.

IHC analysis revealed that Gata3 depleted tumors displayed significantly less Ki67 and more p18 positive cells than control tumors (Figure 5D, E, Figure S6), indicating that Gata3 depleted tumor cells proliferate slower than control cells *in vivo*. The observation that Gata3 depletion in luminal tumor cells induces p18 with reduction of cell proliferation and tumor growth is consistent with our previous finding that p18 is a downstream target of GATA3 restraining luminal cell proliferation and tumorigenesis. These results also suggest that induction of p18 and reduction of cell proliferation by depletion of Gata3 in luminal tumor cells are responsible for decreased tumor growth, even though Gata3 deficiency promotes luminal-to-basal differentiation.

### GATA3 and basal marker expression levels are inversely related in human basal-like breast cancers

**Figure 6:**
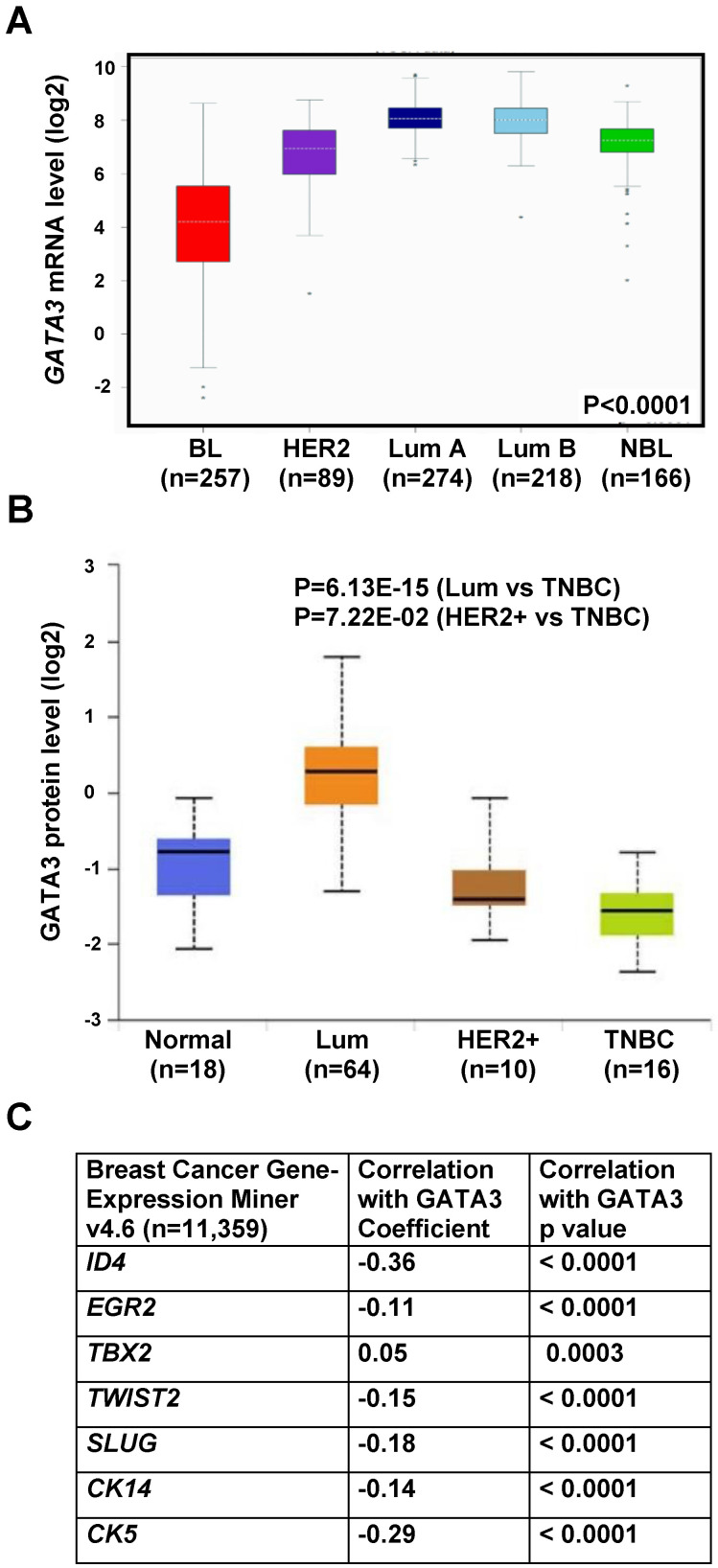
** Correlation analysis of GATA3 with basal markers and subtypes in human breast cancers.** (A) Analysis of *GATA3* mRNA expression in TCGA breast cancer patient samples according to molecular tumor subtype. (B) Analysis of GATA3 protein levels in the Clinical Proteomic Tumor Analysis Consortium (CPTAC) breast cancer dataset according to major subclass (http://ualcan.path.uab.edu/index.html). (C) Correlation analysis of mRNA expression of *GATA3* and basal markers in human breast cancer gene expression miner v4.6 dataset (http://bcgenex.ico.unicancer.fr/BC-GEM/GEM-requete.php).

To determine whether our murine tumors model human breast cancers, we queried *GATA3* expression in TCGA breast cancer patient sample sets 48. We found that *GATA3* mRNA was highly correlated with breast tumor intrinsic subtypes. Specifically, *GATA3* mRNA was significantly low in basal-like tumors and high in luminal A and B tumors (Figure 6A), which is consistent with our previous analysis in the NKI breast cancer patient sample dataset 27. Since basal-like breast cancer accounts for approximately 70% of TNBCs, we then analyzed GATA3 protein levels in the Clinical Proteomic Tumor Analysis Consortium (CPTAC) breast cancer patient sample set and *GATA3* mRNA levels in TCGA breast cancer patient samples 47, 48 according to major clinical subclass. We found that GATA3 expression was significantly low in TNBC and high in luminal tumors (Figure 6B and Figure S7B). Correlation analysis revealed a significant inverse correlation between mRNA expression of *GATA3* with *ID4*, *TWIST2*, *SLUG*, *CK14* and *CK5*, all genes associated with basal differentiation (Figure 6C and Figure S7A). These clinical findings, consistent with our results in mice, further confirm that loss of *GATA3* promotes basal-like cancer development and progression.

## Discussion

In the present study, we demonstrate that haploid loss of *Gata3* reduces MEC proliferation with induction of p18, impairs luminal differentiation, but promotes basal differentiation in mammary development. p18 deficiency rescues the proliferative defect caused by haploid loss of *Gata3* and induces luminal type mammary tumors. Haploid loss of *Gata3* by heterozygous germline deletion of *Gata3*, although insufficient to induce mammary tumors alone, changes the properties of mammary tumors induced by p18 deficiency, resulting in their malignant and luminal-to-basal-like transformation. By investigating MMTV-PyMT mouse mammary tumors, we found that expression of *Gata3* negatively correlates with basal differentiation markers in tumor cells. We generated a Gata3 positive luminal type tumor model system and discovered that depletion of Gata3 in luminal tumor cells reduces cell proliferation with induction of p18, but promotes basal-like differentiation. We further confirmed that GATA3 and basal marker expression levels are inversely correlated in human basal-like breast cancers. This study provides the first genetic evidence demonstrating that loss-of-function of *GATA3* induces basal-like breast cancer. Furthermore, our finding suggests that basal-like breast cancer may also originate from luminal type cancers.

A challenge in investigating the function of GATA3 *in vivo* is that depletion of Gata3 in mice results in early lethality, proliferative defects, or apoptosis 18-21, 32, preventing the determination of its loss-of-function role in controlling cell fate in tumor development and progression. We and other groups identified a few tumor suppressors, p18 and caspase 14 27, 36, 40, as well as oncogenes, cyclin D and c-Myc 39, 55, as critical targets of GATA3 in controlling mammary or lymphoid cell proliferation. Of the identified candidates, knockdown of p18 or overexpression of cyclin D has been reported to rescue proliferative defects induced by GATA3 knockdown in T cells or breast cancer cells *in vitro* 40, 41, 55. However, whether these candidates play a critical role *in vivo* in facilitating GATA3 defective cell proliferation and differentiation in tumorigenesis remains to be investigated. We previously reported that p18 is a downstream target of GATA3 and restrains luminal progenitor cell proliferation 27, the findings presented in this paper provide genetic evidence suggesting that loss-of-function of Gata3 results in accumulation of p18 in luminal progenitor cells, blocking them from entering an active cell cycle and undergoing subsequent aberrant basal differentiation. Loss of p18 stimulates proliferation of luminal progenitor cells and initiates luminal tumorigenesis with minimal impairment of luminal lineage differentiation in the presence of functional Gata3. Our results demonstrating that p18 depletion rescues the proliferative defects induced by haploid loss of *Gata3* and that *p18*;*Gata3* double mutant mice develop basal-like mammary tumors suggest that depletion of p18 is required for proliferation of GATA3 defective cells and for development of GATA3 deficient basal-like mammary tumors.

Consistent with the findings derived from mice, in human breast cancers, loss of p18 and amplification or overexpression of cyclin D and CDK4 are frequently detected in luminal type tumors whereas loss of GATA3 expression and loss or mutation of *Rb* are key features of BLBCs 8, 27-31. In addition to the results from mice showing that loss of p18 alone or loss of both Rb and p107 induces GATA3-positive luminal tumors 27, 43, these findings suggest that loss-of-function of the p18-cyclin D/CDK4-Rb pathway induces luminal tumorigenesis, and loss of Gata3 converts the p18-cyclin D/CDK4-Rb pathway deficient luminal type tumors into basal-like tumors. Interestingly, though loss-of-function of the p18-cyclin D/CDK4-Rb pathway is a common event in both luminal and basal-like breast cancers, loss of or mutation of *Rb* per se is mainly detected in BLBCs, which also explains why clinically defined luminal type tumors, but not basal-like tumors, are more sensitive to CDK4 inhibitors since CDK4 promotes cell proliferation dependent on functional RB.

The function of GATA3 in suppressing breast tumor development, metastasis, and EMT has been well studied by overexpressing GATA3 in cell line models 33, 34, 56, 57. Two independent groups investigated the role of loss-of-function of Gata3 in mammary tumor development and progression in MMTV-PyMT transgenic mice 35, 36. Heterozygous germline mutation of *Gata3* in MMTV-PyMT transgenic mice accelerates mammary tumor onset 36 and loss of Gata3 marks malignant progression in MMTV-PyMT mammary tumors 35. However, due to growth defects induced by long-term loss of *Gata3* and apoptosis caused by acute loss of *Gata3* in differentiated tumor cells 27, 35, 40, it remains elusive if *Gata3* loss promotes basal differentiation in breast cancer development and progression. In the present study, we found that haploid loss of *Gata3* by heterozygous germline deletion impaired luminal but activated basal differentiation in mammary epithelial and cancerous cells. Depletion of Gata3 in MMTV-PyMT luminal tumor cells further confirmed the activation of basal and impairment of luminal differentiation. These results provide compelling genetic evidence suggesting that in addition to inactivation of luminal differentiation, loss of Gata3 promotes basal-like differentiation in mammary and tumor development and progression.

It has long been suggested that BLBCs originate from mammary basal epithelial cells. Recently, a few groups demonstrated that *BRCA1* mutant BLBCs may originate from aberrant luminal progenitor cells 14-16. In our previous study, we discovered that p18 deficiency stimulates luminal progenitor cell proliferation and induces luminal mammary tumors, and that germline deletion of *Brca1* impairs luminal but activates basal differentiation of p18 deficient luminal progenitor cells, eventually leading to development of BLBC 17, 27. These findings confirm that the aberrantly differentiated luminal epithelial cells are the origin of BLBCs developed in mice carrying heterozygous germline mutation of *Brca1*. In the present study, we utilized a similar mouse model system to investigate the role of haploid loss of *Gata3* in mammary cell differentiation. To avoid aberrant differentiation caused by artificially choosing distinct cre transgenic mice and directing *Gata3* deletion in specific cell lineages in conditional *Gata3*^f/f^ mice, we analyzed mice harboring heterozygous germline deletion of *Gata3* which enables us to investigate the role of haploid of *Gata3* in all cell linages in an unbiased manner. We demonstrated that heterozygous germline deletion of *Gata3* also impairs luminal but activates basal differentiation of p18 deficient luminal progenitor cells, which eventually lead to development of BLBCs. Furthermore, we discovered that depletion of *Gata3* in MMTV-PyMT luminal tumor cells promotes basal differentiation and leads to development of BLBCs. These findings not only confirm the function of Gata3 loss in promoting basal-like differentiation in mammary epithelial and cancerous cells, but also suggest the luminal cell origin of non-*BRCA1* mutant BLBCs.

## Conclusions

This study provides the first genetic evidence demonstrating that loss-of-function of GATA3 directly induces basal-like breast cancer. Furthermore, our finding suggests that basal-like breast cancer may also originate from luminal type cancers.

## Supplementary Material

Supplementary figures and table.

## Figures and Tables

**Table 1:** Spontaneous mammary tumor development in p18 and Gata3 mutant mice ^a^

Genotype	Tumor		
	Mammary tumor	Luminal Marker+ tumor ^d^	Basal Marker+ tumor ^e^
WT	0/9		
*p18^mt b^*	8/27 (30%)	6/8 (75%)	2/8 (25%)
*Gata3* ^+/-^	0/8		
*p18*^mt^;*Gata3*^+/- c^	17/34 (50%)^f^	3/17 (18%)^g^	14/17 (82%)^g, h^

**a** All mice were in Balb/c-B6 mixed background and were at 8-22 months of age. **b** This group contains eight *p18^+/-^* and nineteen *p18^-/-^* mice. **c** This group contains ten *p18*^+/-^;*Gata3*^+/-^ and twenty four *p18*^-/-^;*Gata3*^+/-^ mice. **d** ER was detected in >2% tumor cells and Ck8 or E-Cad were detected in >50% tumor cells by IHC or IF. **e** At least one of the basal markers (Ck5, Ck14, and Sma) was positively detected in >2% tumor cells by IHC or IF, as we previously reported (Bai, Oncogene, 2013; Cancer Res., 2014). **f** No significance from *p18*^mt^;*Gata3*^+/-^ and *p18*^mt^ tumors by a two-tailed Fisher's exact test (p=0.124). **g** Significance from *p18*^mt^;*Gata3*^+/-^ and *p18*^mt^ tumors by a two-tailed Fisher's exact test (p=0.010). **h** Five mammary tumors metastasized to lung and/or liver.

## Abbreviations

p18: p18^INK4c^
MaSC: mammary stem cell
HER2: human epidermal growth factor receptor 2
ER: estrogen receptor
PGR: progesterone receptor
TNBC: triple-negative breast cancer
BLBC: basal-like breast cancer
HDR: hypoparathyroidism-deafness-renal disease
HSC: hematopoietic stem cell
MEC: mammary epithelial cell
NCG: NOD-Prkdcem26Cd52Il2rgem26Cd22/Nju
IHC: immunohistochemistry
E-Cad: E-cadherin
KD: knockdown
MFP: mammary fat pad
LP: luminal progenitor
