# Supplementary material for: Loss of function of GATA3 induces basal-like mammary tumors
Source: Theranostics. 2022 Jan 1;12(2):720–33. doi: 10.7150/thno.65796 (PMC8692904; doi:10.7150/thno.65796)
Supplement: Supplementary file 1 — Supplementary figures and table. [file thnov12p0720s1.pdf]

Table S1:

List of primer sequence

Gene	Species	FW/RV	Sequence
Gapdh	Mouse	FW	GGTGAAGGTCGGTGTGAACG
Gapdh	Mouse	RV	TGTAGACCATGTAGTTGAGG
Gata3	Mouse	FW	GCTGACGGAAGAGGTGGACGTACT
Gata3	Mouse	RV	TGGTGGGTCGGAGGATACCT
Cdh1	Mouse	FW	AACAACTGCATGAAGGCGGGAATC
Cdh1	Mouse	RV	CCTGTGCAGCTGGCTCAAATCAAA
Foxa1	Mouse	FW	CAGGGTTGGATGGTTGTGTC
Foxa1	Mouse	RV	GTTGCTGACAGGGACAGAGG
p18	Mouse	FW	GAGCCTTGGGGGAACGAGTTG
p18	Mouse	RV	TCTCCGGATTTCCAAGTTTC
Egr2	Mouse	FW	GCCAAGGCCGTAGACAAAATC
Egr2	Mouse	RV	CCACTCCGTTCATCTGGTCA
Id4	Mouse	FW	CAGTGCGATATGAACGACTGC
Id4	Mouse	RV	GACTTTCTTGTTGGGCGGGAT
Tbx2	Mouse	FW	CCGATGACTGCCGCTATAAGT
Tbx2	Mouse	RV	CCATCCACTGTTCCCCTGT
Slug	Mouse	FW	CACATTCGAACCCACACATTGCCT
Slug	Mouse	RV	TGTGCCCTCAGGTTTGATCTGTCT
Sma	Mouse	FW	GAGAAGCCCAGCCAGTCG
Sma	Mouse	RV	CTCTTGCTCTGGGCTTCA
Esr1	Mouse	FW	GCAAGTGTTACGAAGTGGGCATG
Esr1	Mouse	RV	GCAGCCCTCATGTCTCCTGAAG
GATA3	Human	FW	CACAACCACACTCTGGAGGAG
GATA3	Human	RV	GTCCTCCAGTGAGTCATGCAC
CDH1	Human	FW	CCAGGAGCCAGACACATTTATGG
CDH1	Human	RV	CTGTGTACGTGCTGTTCTTCACG
ESR1	Human	FW	CGGCTCCGTAAATGCTACGAAGT
ESR1	Human	RV	CTCTCTGGCGCTTGTGTTTCAAC
TWIST2	Human	FW	GTCCATGTCCGCCTCCCACT
TWIST2	Human	RV	TCTCTCGACGCTGGTGGAGG
EGR2	Human	FW	TCAACATTGACATGACTGGAGAG
EGR2	Human	RV	AGTGAAGGTCTGGTTTCTAGGT
SMA	Human	FW	AAAAGACAGCTACGTGGGTGA
SMA	Human	RV	GCCATGTTCTATCGGGTACTTC
SLUG	Human	FW	CCCACACATTACCTTGTGTTTGCAA
SLUG	Human	RV	CAAATGCTCTGTTGCAGTGAGG
GAPDH	Human	FW	AGGTGAAGGTCGGAGTCAAC
GAPDH	Human	RV	AGTTGAGGTCAATGAAGGGG
Sh-Gata3-a	Mouse		gatccgGTACATGGAAGCTCAGTATCCTCAAGA GGGATACTGAGCTTCCATGTACtttttg
Sh-Gata3-b	Mouse		gatccgGCCAGATAGCATGAAGCTGGATCAAGA GTCCAGCTTCATGCTATCTGGCtttttg
Sh-Gata3-c	Mouse		gatccgGGCTGTACTACAAGCTTCATATCAAGA GTATGAAGCTTGTAGTACAGCCtttttg
Sh-control	Mouse		gatccgACAGAAGCGATTGTTGATCtttttg

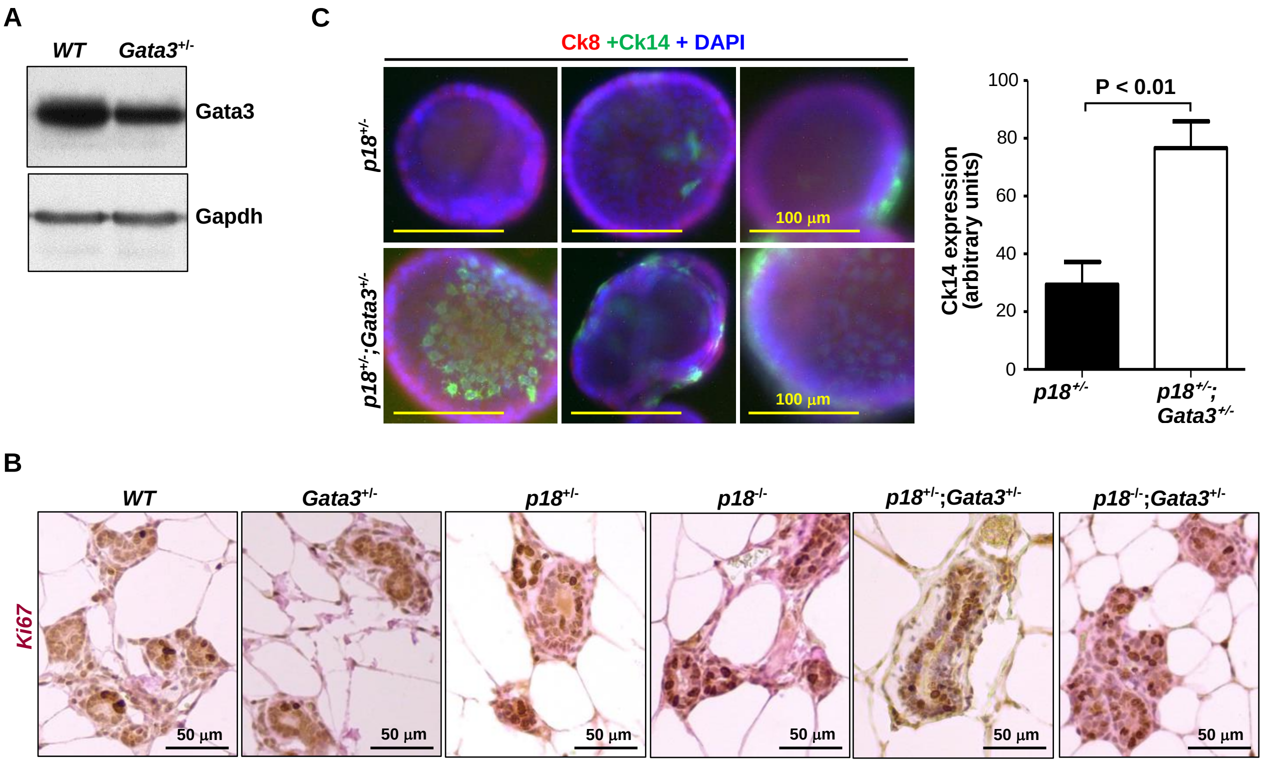

Figure S1. Analysis of Gata3 heterozygous mice and cells. (A) Gata3 heterozygosity leads to reduction of Gata3 expression in spleen. Tissues from spleen of WT and Gata3$^{+/-}$ mice at 2 months of age were analyzed. (B) Gata3 heterozygosity reduces MEC proliferation, which is rescued by p18 deficiency. Representative mammary tissue from 8-10-month old mice were analyzed by immunohistochemistry with Ki67. (C) Gata3 heterozygosity promotes basal differentiation in MECs. Freshly isolated mammary cells were cultured in Matrigel-coated plates. Nine days after culture, the colonies were immunostained with Ck8 and Ck14. Ck14 expression in colonies was quantified by ImageJ software (right panel). The assay was performed in triplicate for each animal. The bar graphs represent the mean ± SD of two animals per group.

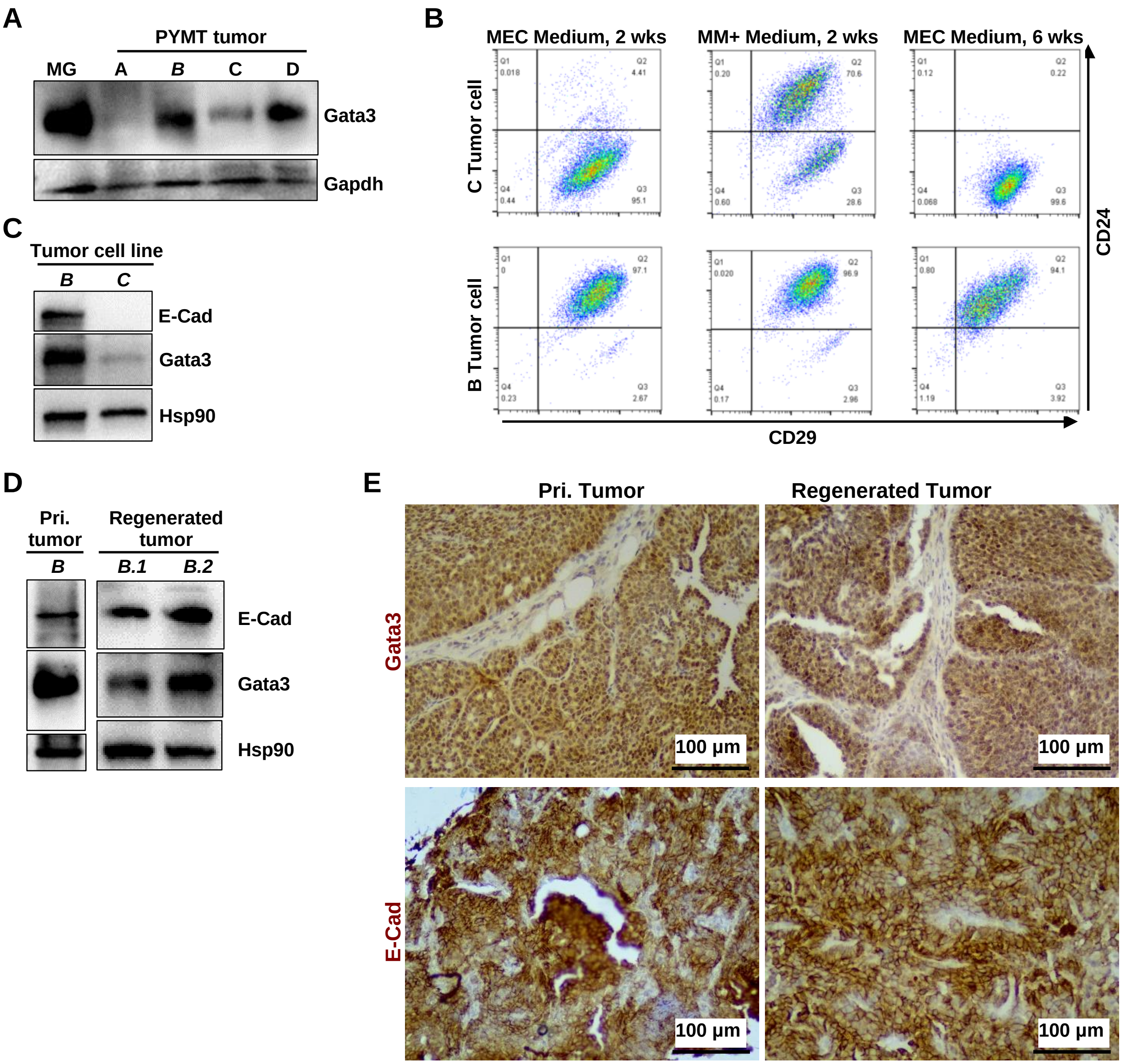

Figure S2. Establishment of a Gata3 functional murine luminal tumor system. (A) Screening of mammary tumors developed in individual MMTV-PyMT mice. MG, mammary gland from WT mouse. Note the distinct level of GATA3 in tumors. (B) FACS analysis of primary tumor cell lines derived from tumor B and C in (A) and cultured in MEC and MM+ media. Note that C tumor cells contained two populations ($CD24^{-}CD29^{low}$ and $CD24^{+}CD29^{low}$) in MM+ medium in two weeks and one $CD24^{-}CD29^{low}$ population in MEC medium in two and six weeks whereas B tumor cells contained only one $CD24^{+}CD29^{low}$ population in both media and all time points. (C) Western blot analysis of primary tumor cell lines derived from tumor B and C in (A) and cultured in MEC media. (D, E) B tumor cell line was transplanted into mammary fat pads (MFPs) of NCG mice and the regenerated tumors were then analyzed by western blot (D) and IHC (E). Note the comparable expression level and pattern of Gata3 and E-Cad between primary and regenerated tumors, as well as among individual tumors regenerated. Pri., primary.

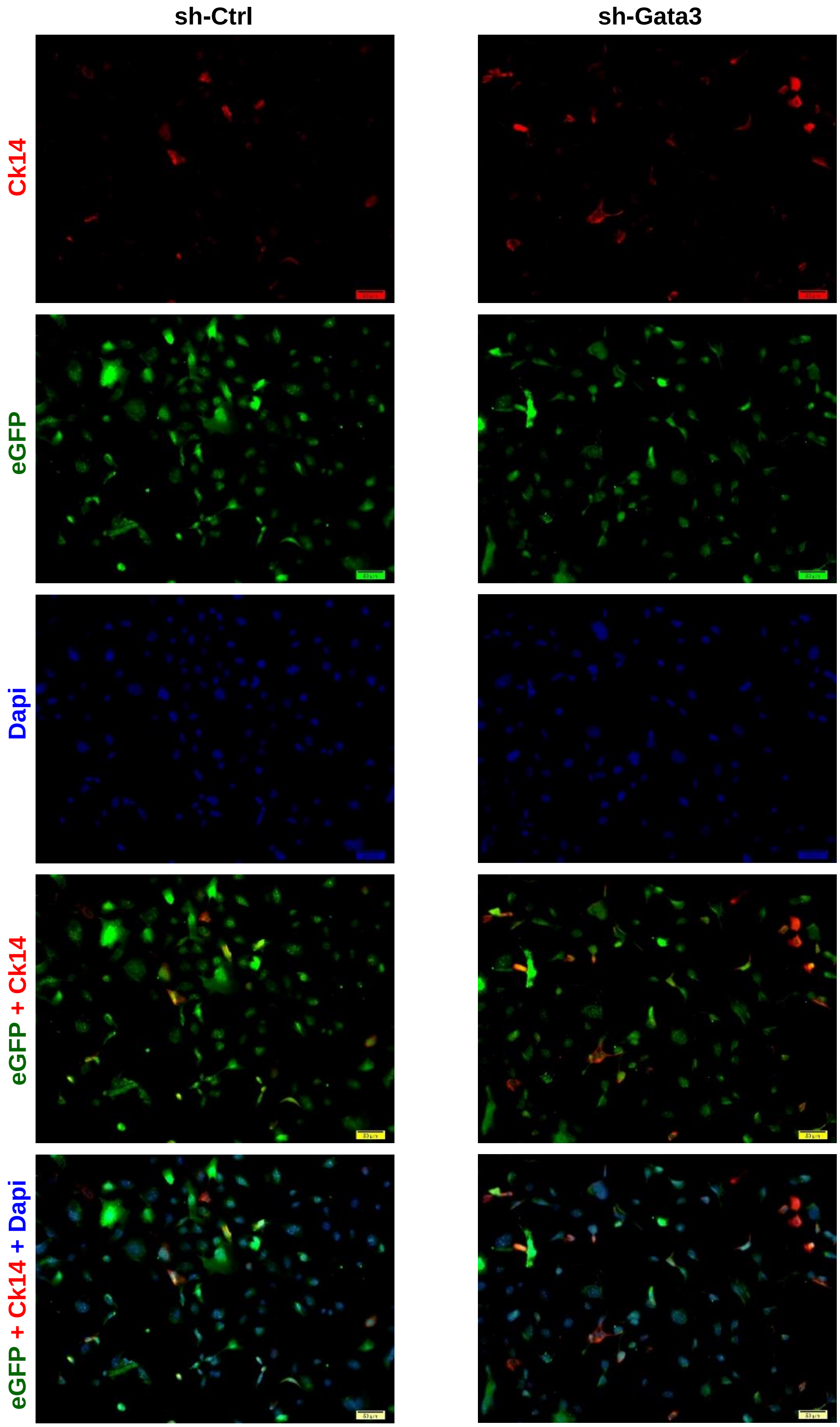

Figure S3. Depletion of Gata3 in luminal tumor cells stimulate basal-like differentiation. Representative immunofluorescent staining analysis of the MMTV-PyMT luminal tumor cells infected with sh-Ctrl and sh-Gata3. Cells were immunostained with antibodies against eGFP (Green) and Ck14 (Red). Note the increased eGFP and Ck14 double positive cells in sh-Gata3 group.

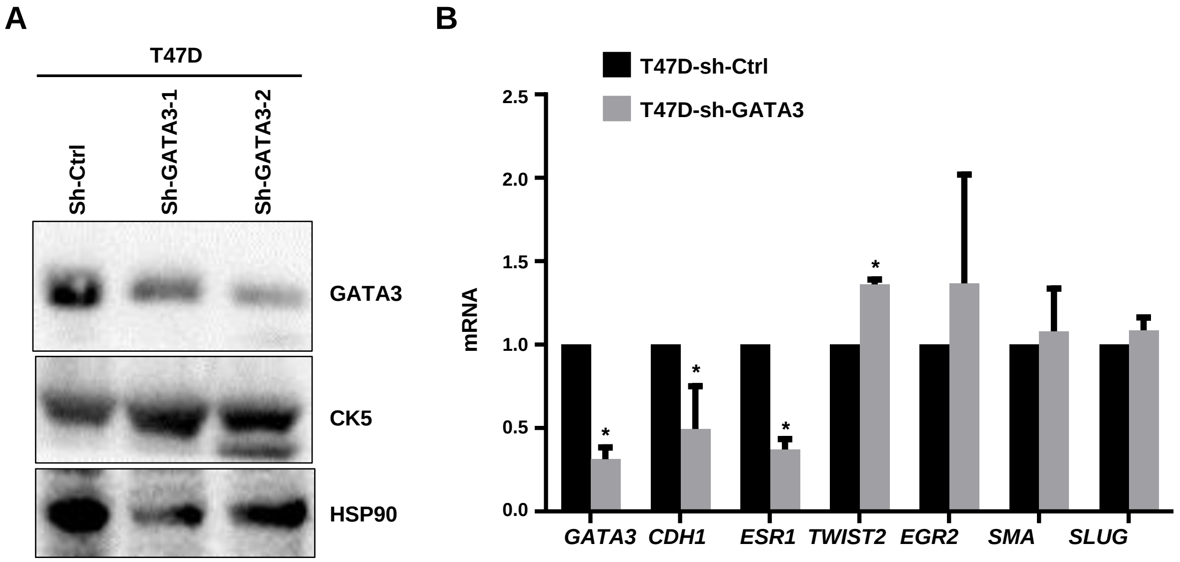

Figure S4. Knockdown of GATA3 in human luminal breast cancer cells significantly reduces the expression of genes associated with luminal differentiation, and moderately enhances the expression of genes associated with basal differentiation. (A, B) T47D cells were infected with pGIPZ-sh-Control (sh-Ctrl) or pGIPZ-sh-GATA3 targeting different sequences of human GATA3 (sh-GATA3-1 and sh-GATA3-2), selected with puromycin, and analyzed by western blot (A) and qRT-PCR (B).

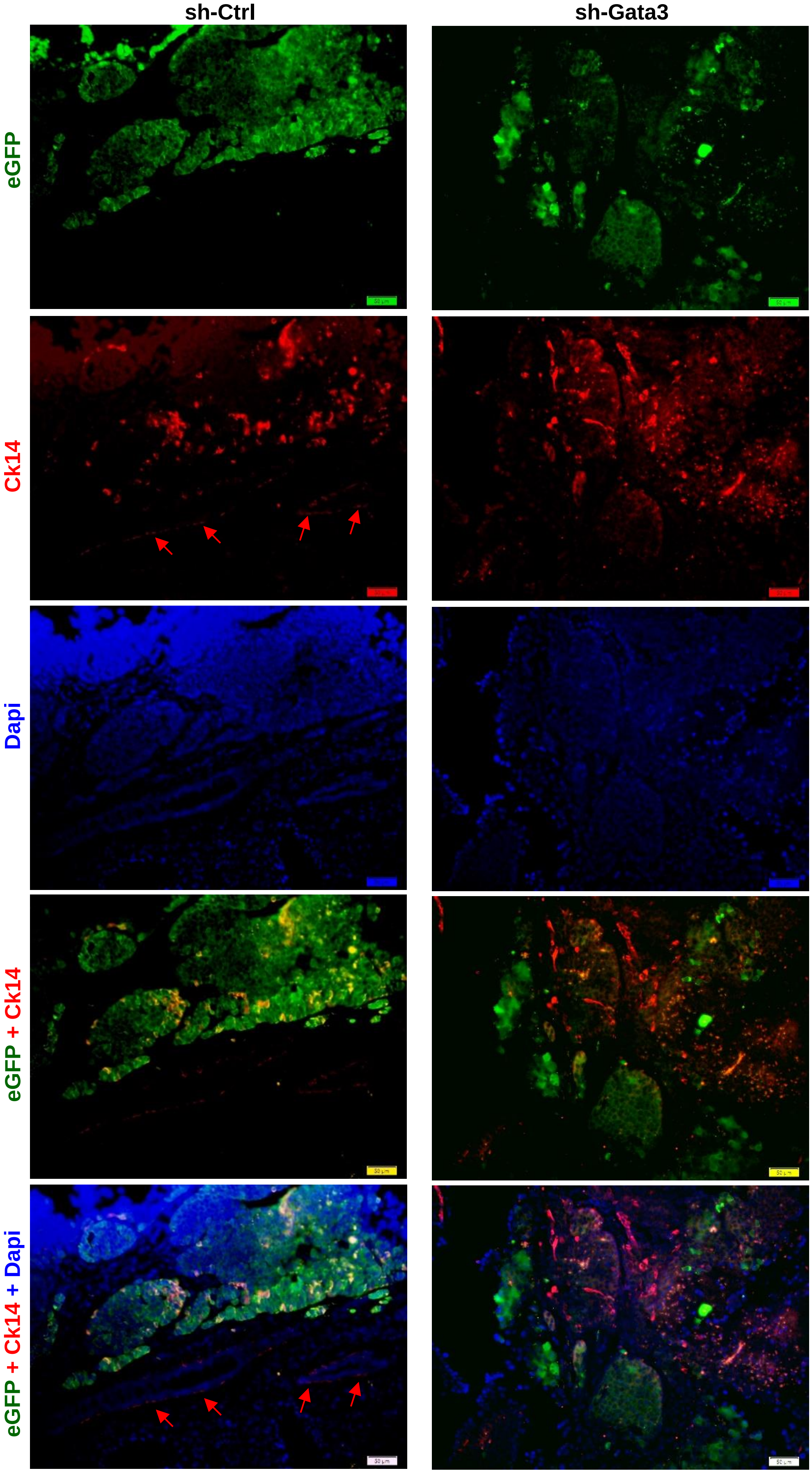
sh-Ctrl
sh-Gata3
eGFP
Ck14
Dapi
eGFP + Ck14
eGFP + Ck14 + Dapi

Figure S5. Depletion of Gata3 in luminal tumor cells induces basal-like tumors in vivo.

Mammary tumors formed by transplantation of MMTV-PyMT luminal tumor cells stably expressing sh-Ctrl or sh-Gata3 were immunostained with antibodies against eGFP and Ck14. Ck14 positive basal cells in tumor-free mammary glands are indicated (Red arrows).

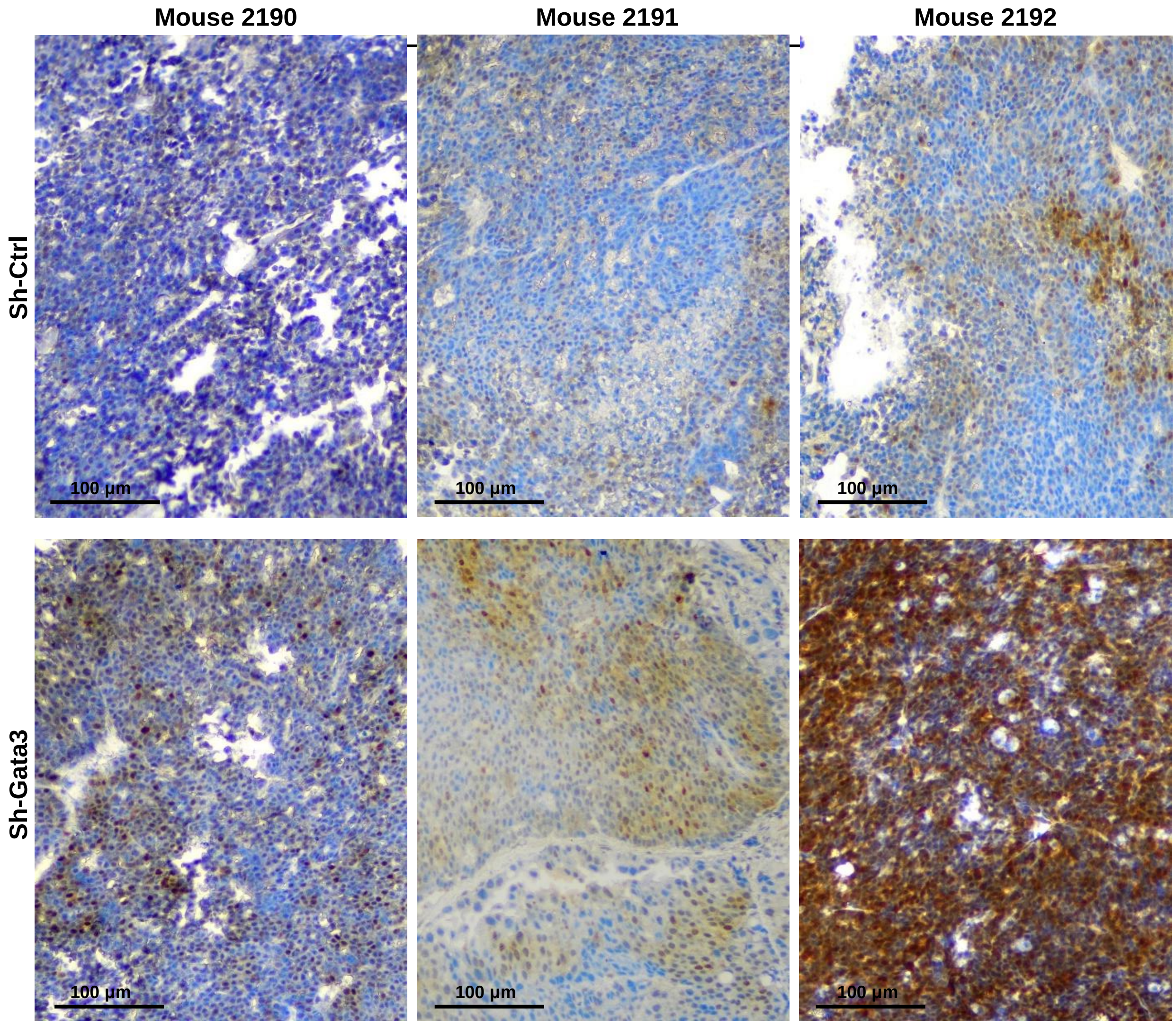

Fig. S6. Depletion of Gata3 in luminal tumor cells stimulates the expression of p18 in regenerated tumors. MMTV-PyMT luminal tumor cells stably expressing sh-Ctrl or sh-Gata3 were transplanted into the left and right inguinal MFPs of NCG mice, respectively. Mammary tumors regenerated were immunostained with antibodies against p18. Note the highly expressed p18 in sh-Gata3 tumors relative to that in sh-Ctrl tumors.

A

GEPIA Breast cancer Database	Correlation with GATA3 Coefficient	Correlation with GATA3 p value
ID4	**-0.35**	**5.1E-33**
EGR2	**-0.084**	**0.0058**
TBX2	**0.13**	**1.2E-05**
TWIST2	**-0.19**	**1.6E-10**
SLUG	**-0.19**	**9.9E-11**
CK14	**-0.24**	**2.1E-15**
CK5	**-0.34**	**1.8E-31**

B

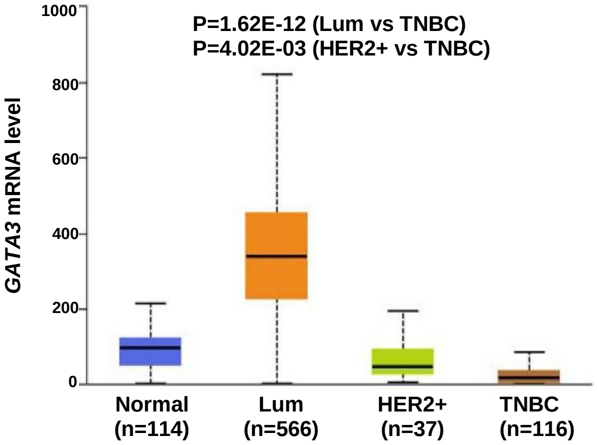

Figure S7. Analysis of GATA3 with basal markers and major subclasses in human breast cancers. (A) Correlation analysis of mRNA expression of GATA3 and basal markers in Gene Expression Profiling Interactive Analysis (GEPIA) human breast cancer dataset (http://gepia.cancer-pku.cn/). (B) Analysis of *GATA3* mRNA expression in TCGA breast cancer patients according to major subclass in UALCAN dataset (http://ualcan.path.uab.edu/index.html).
